# Clinical features and fecal microbiota characteristics of patients with both ulcerative colitis and axial spondyloarthritis

**DOI:** 10.1186/s12876-024-03150-w

**Published:** 2024-01-31

**Authors:** Lei Zhangni, Xiao Mofan, Chen Yuling, Li Yingchao

**Affiliations:** Department of Gastroenterology, the First Affiliated Hospital of Xi’an Jiao Tong University, Xi’an, 710061 China

**Keywords:** Ulcerative colitis, Axial spondyloarthritis, Fecal microbiota, 16S rDNA sequencing

## Abstract

**Background:**

The role of the intestinal microbiota in the pathogenesis of inflammatory bowel disease combined with axial spondyloarthritis (axSpA) is gaining widespread interest.

**Aims:**

This study was conducted to investigate the clinical and fecal microbiota characteristics of patients with both ulcerative colitis (UC) and axSpA.

**Methods:**

Clinical data were collected from patients with UC. Patients were divided into the axSpA and non-axSpA groups according to human leukocyte antigen-B27 serology and sacroiliac joint imaging results. We obtained fecal specimens from 14 axSpA and 26 non-axSpA patients. All samples underwent 16S ribosomal DNA sequencing.

**Results:**

Seventy-three patients with UC were included in this study, and the axSpA incidence was 19.2%. This incidence was significantly higher in patients with C-reactive protein > 10 mg/L. Firmicutes and *Faecalibacterium* abundances were decreased, and Proteobacteria and *Escherichia_Shigella* abundances were increased in the axSpA group compared with those of the non-axSpA group. Indicator analysis showed that *Escherichia_Shigella* was more likely to be an indicator species of axSpA. Additionally, many biosynthetic and metabolic pathways, including glutathione metabolism, fatty acid degradation, geraniol degradation, and biosynthesis of siderophore group nonribosomal peptides, were upregulated in the axSpA group.

**Conclusion:**

Patients with UC have a high axSpA incidence, which may be related to the relative abundances of *Escherichia_Shigella* in these patients. The abundances of various biosynthetic and metabolic pathways of the fecal flora were upregulated in patients with axSpA.

**Supplementary Information:**

The online version contains supplementary material available at 10.1186/s12876-024-03150-w.

## Introduction

Inflammatory bowel disease (IBD) is an incurable chronic inflammatory disorder of the gastrointestinal tract that mainly includes ulcerative colitis (UC) and Crohn’s disease. In addition to typical intestinal manifestations, IBD can involve multiple organs and tissues throughout the body, including the skin, eyes, joints, liver, lungs, and/or pancreas. Axial spondyloarthritis (axSpA) is a group of chronic inflammatory diseases mainly affecting the spine, including ankylosing spondylitis (AS) and non-radiographic axSpA (nraxSpA) [1]. nraxSpA differs from axSpA in its lack of radiographically confirmed sacroiliitis. IBD and axSpA are closely associated with each other [2, 3]. axSpA occurs in 3%–25% of patients with IBD and is among the most frequent extraintestinal manifestations in these individuals [4–6]. Approximately half of patients with axSpA experience gut inflammation, and axSpA has been linked to disease activity underscoring the effect of gut inflammation in axSpA [7, 8].

Two theories, including alterations in gut bacteria and migration of gut lymphocytes to the joints, may explain axSpA development in patients with IBD [9, 10]. Changes in the gut bacteria, known as dysbiosis, have been associated with UC-axSpA. However, few data have been reported on the fecal flora structure in patients with IBD-axSpA. Our study was conducted to explore possible associations between the fecal bacterial characteristics and occurrence of axSpA in patients with UC to provide new insight into the underlying pathogenetic mechanisms suggested to link these two entities and to guide treatment decisions.

## Materials and methods

### Patients and study design

Patients with UC who were hospitalized at the Department of Gastroenterology at the First Affiliated Hospital of Xi’an Jiaotong University from October 2020 to June 2022 were recruited. The IBD diagnoses were confirmed by gastroenterologists and further confirmed by endoscopy and pathology. Patients were aged between 20 and 70 years, and all volunteered to participate. Exclusion criteria were receipt of antibiotics or probiotics within 3 months prior to study enrollment, prior treatment with any immunosuppressive agent, refusal to undergo relevant tests for a definitive diagnosis, and/or suffering from any acute or chronic cardiovascular, gastrointestinal, or immunological condition.

Patients with UC presenting with inflammatory low back pain for ≥3 months and were <45 years old were evaluated based on either clinical findings per the judgment of the treating rheumatologist or imaging findings on magnetic resonance imaging or radiography. The patients were divided into either the axSpA or non-axSpA group. Patients in the axSpA group met the Assessment of Spondyloarthritis International Society criteria for axSpA [11]. The Ethics Committee of the First Affiliated Hospital of Xi’an Jiaotong University School approved the study methodology (No. XJTU1AF2022LSK-146).

### Clinical data collection

We collected data on patients’ sex, body mass index (BMI), smoking history, UC onset time, disease staging, clinical typing, Montreal classification of extent, modified Truelove and Witts classification, and modified Mayo score. Human leukocyte antigen (HLA)-B27 status was determined via flow cytometry. Laboratory tests for C-reactive protein (CRP) and erythrocyte sedimentation rate (ESR) were used to objectively evaluate disease activity. Immunoturbidimetric assay was used to measure CRP concentration in blood drawn after fasting on the second day of admission.

### Fecal sample processing

After signing informed consent, patients provided ≥5 g of fresh stool sample for sample collection before the use of any therapeutic medications. Samples were placed in numbered, sterile 5-mL freezing tubes, then transferred to our biospecimen bank within 2 hours and stored in liquid nitrogen quick-freezing at −80°C until processed. Fourteen specimens were retained from the axSpA group, and 26 were retained from the non-axSpA group.

### 16S ribosomal DNA sequencing and fecal specimen analysis

Microbial DNA was extracted using HiPure Stool DNA Kits (Magen, Guangzhou, China) per the manufacturer’s protocols. The full-length 16S rDNA was amplified by PCR (94°C for 2 min, followed by 30 cycles of 98°C for 10 s, 65°C for 30 s, and 68°C for 30 s, with a final extension at 68°C for 5 min) using primers 341F: CCTACGGGGNGGCWGCAG and 806R: GGACTACHVGGGTATCTAAT. The 50-μL mixture contained 5 μL of 10× buffer KOD, 5 μL of 2 mM dNTPs, 3 μL of 25 mM MgSO_4_, 1.5 μL of primer F (10 μM), 1.5 μL of primer R (10 μM), 1 μL of KOD DNA polymerase, and 100 ng of template DNA. Related PCR reagents were from New England Biolabs, Inc. (Ipswich, MA, USA). Amplicons were extracted from 2% agarose gels and purified using the AxyPrep DNA Gel Extraction Kit (Axygen Biosciences, Union City, CA, USA) per the manufacturer’s instructions and quantified using the ABI StepOnePlus Real-Time PCR System (Life Technologies, Foster City, CA, USA). Purified amplicons were pooled in equimolar amounts and paired-end sequenced (PE250) on an Illumina platform according to standard protocols.

### Statistical analysis

All statistical analyses were performed using SPSS software, version 25.0, for Windows (IBM Corporation, Armonk, New York, NY, USA). Normally distributed continuous variables are expressed as means ± standard deviation. Non-normally distributed continuous variables are expressed as the median (quartile) (M [Q]). Categorical variables are expressed as the constituent ratio or rate (%). For normally distributed continuous variables, t-tests were used if the variance was homogeneous, and the t’ test was used if the variance was not homogeneous. For non-normally distributed continuous variables, the Mann-Whitney U test was used. Categorical variables were compared between groups using chi-square tests.

Bioinformatic analysis was performed using Omicsmart (Guangzhou City, Guangdong Province, China), a real-time interactive online platform for data analysis. Stacked bar plots of the community compositions were constructed using the R project ggplot2 package (version 2.2.1). Kyoto Encyclopedia of Genes and Genomes (KEGG) pathway analysis of the operational taxonomic units (OTUs) was inferred using PICRUSt (version 2.1.4). Alpha index comparisons, species comparisons, and functional differences between groups were calculated using Welch’s t-test. *P*<0.05 was considered statistically significant.

## Results

### Univariate analysis

Seventy-three patients with UC were enrolled: 37 (50.7%) had chronic back pain (back pain >3 months), and 14 (19.2%) had axSpA. Of these, 6 with axSpA (42.9%) were diagnosed after the IBD diagnosis, 5 (35.7%) were diagnosed with axSpA at the same time as UC, and 3 (21.4%) were diagnosed with axSpA before being diagnosed with UC. The HLA-B27-positive rate in the axSpA group was 42.9% (6/14). Table 1 compares the baseline demographics and clinical data between the axSpA and non-axSpA groups (Table 1 on pages 21-22, lines 470-471). The proportion of patients with CRP >10 mg/L was larger in the axSpA group than in the non-axSpA group (57.1% vs. 16.9%, *P*=0.005). Sex, BMI, smoking history, UC onset time, clinical typing, Montreal classification of extent, modified Truelove and Witts severity classification, modified Mayo score, proportion of ESR increase, and medication history (5-amino salicylic acids, infliximab, and vedolizumab) did not significantly differ between the groups (*P*>0.05).

**Table 1:** Demographic and clinical characteristics of patients with UC

	axSpA group (*n*_*1*_ = 14)	non-axSpA group (*n*_*2*_ = 59)	*t/χ*^*2*^*/Z*	*P* value
Male (%)	9 (64.3)	39 (66.1)	0.000	1.000
BMI, kg/m^2^	21.06 ± 2.60	21.53 ± 2.93	0.549	0.585
Smoking history, y	4 (28.6)	12 (20.3)	0.096	0.756
UC onset time, y	37.71 ± 9.20	42.39 ± 14.57	1.506	0.142
HLA-B27 +	6 (42.9%)	Not measured		
Disease staging			-	1.000
Active phase	13 (92.9)	55 (93.2)		
Remission	1 (7.1)	4 (6.8)		
Clinical typing				
Initial	2 (14.3)	7 (11.9)	0.000	1.000
Chronic recurrent	12 (85.7)	52 (88.1)		
Montreal classification of extent			2.638	0.267
E1 (proctitis)	1 (7.1)	13 (22.0)		
E2 (left-sided; distal)	4 (28.6)	21 (35.6)		
E3 (pancolitis)	9 (64.3)	25 (42.4)		
Modified Truelove Witts classification			4.369	0.113
Mild	4 (28.6)	16 (18.6)		
Moderate	4 (28.6)	32 (54.2)		
Severe	6 (42.9)	11 (27.1)		
Modified Mayo score	8.43 ± 1.87	7.15 ± 2.27	-1.947	0.055
ESR > 20 mm/h	7 (50.0)	19 (32.2)	0.883	0.347
CRP > 10 mg/L	8 (57.1)	10 (16.9)	7.795	0.005
Medication History				
5-Amino salicylic acids	9 (64.3)	44 (74.6)	0.196	0.658
Infliximab	2 (14.3)	6 (10.2)	0.000	1.000
Vedolizumab	0 (0.0)	5 (8.5)	-	0.576

### Characterization of fecal flora

#### Diversity analysis

**Fig. 1:**
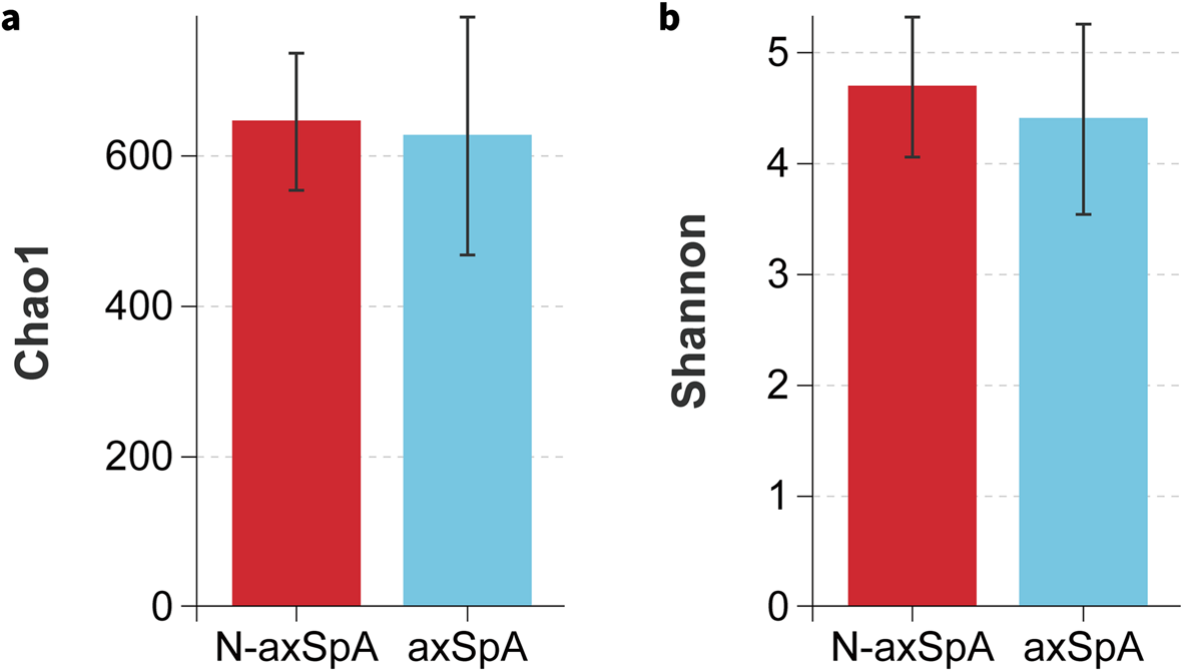
Alpha diversities of the fecal flora in patients with UC

In total, 5011145 16S rRNA reads were generated from fecal samples provided by the axSpA and non-axSpA groups, with averages of 127715±7705 reads per axSpA patient and 123967±12575 reads per non-axSpA patient. In both cases, rarefaction curves established that extra sampling would be of limited benefit (Online Supplementary Fig. S1). Alpha diversity indexes of richness and evenness were assessed with the Chao1 and Shannon indexes, respectively, and visualized using bar graphs (Fig. 1). The alpha diversities did not differ significantly between the groups (Chao1: *P*=0.683, Shannon: *P*=0.279).

**Fig. 2:**
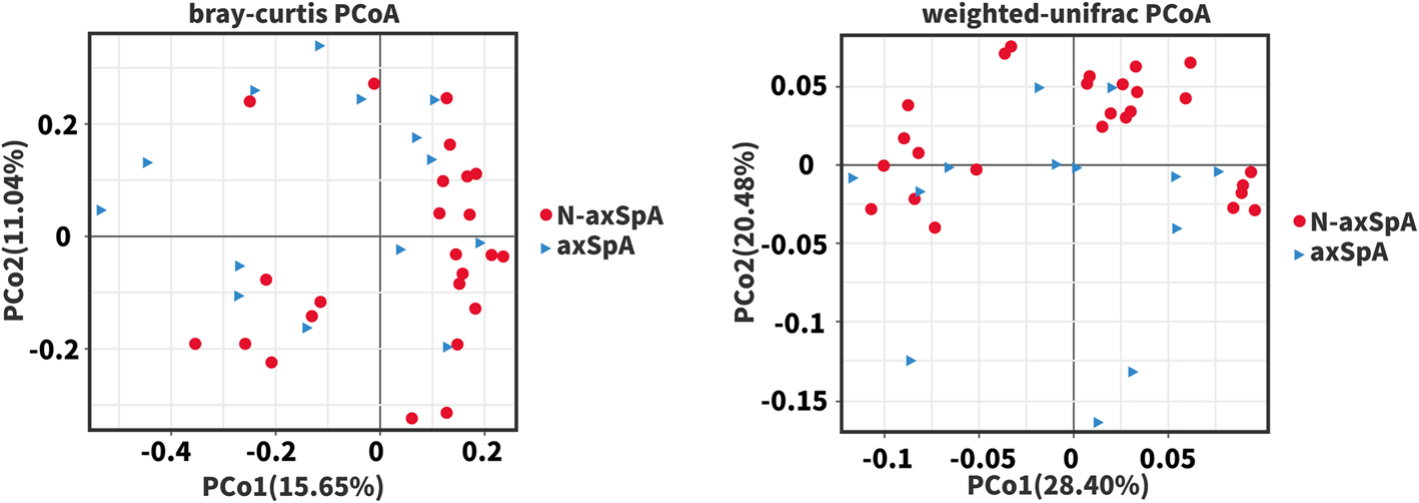
PCoA of the fecal flora in patients with UC

**Fig. 3:**
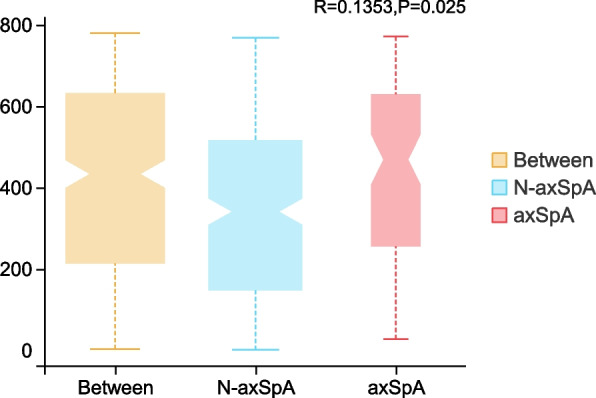
ANOSIM analysis of the fecal flora in patients with UC

Beta diversity was assessed with the Bray-Curtis and weighted-UniFrac measures and visualized using principal coordinates analysis (PCoA). Blue triangles and red dots reflect axSpA patients and non-axSpA patients, respectively. PCoA of the 40 patients demonstrated that the microbial populations of the axSpA patients were clustered away from those of the non-axSpA patients (Fig. 2). This clustering was confirmed by the ANOSIM test (*R*=0.135, *P*=0.026; Fig. 3).

#### Community composition analysis

**Fig. 4:**
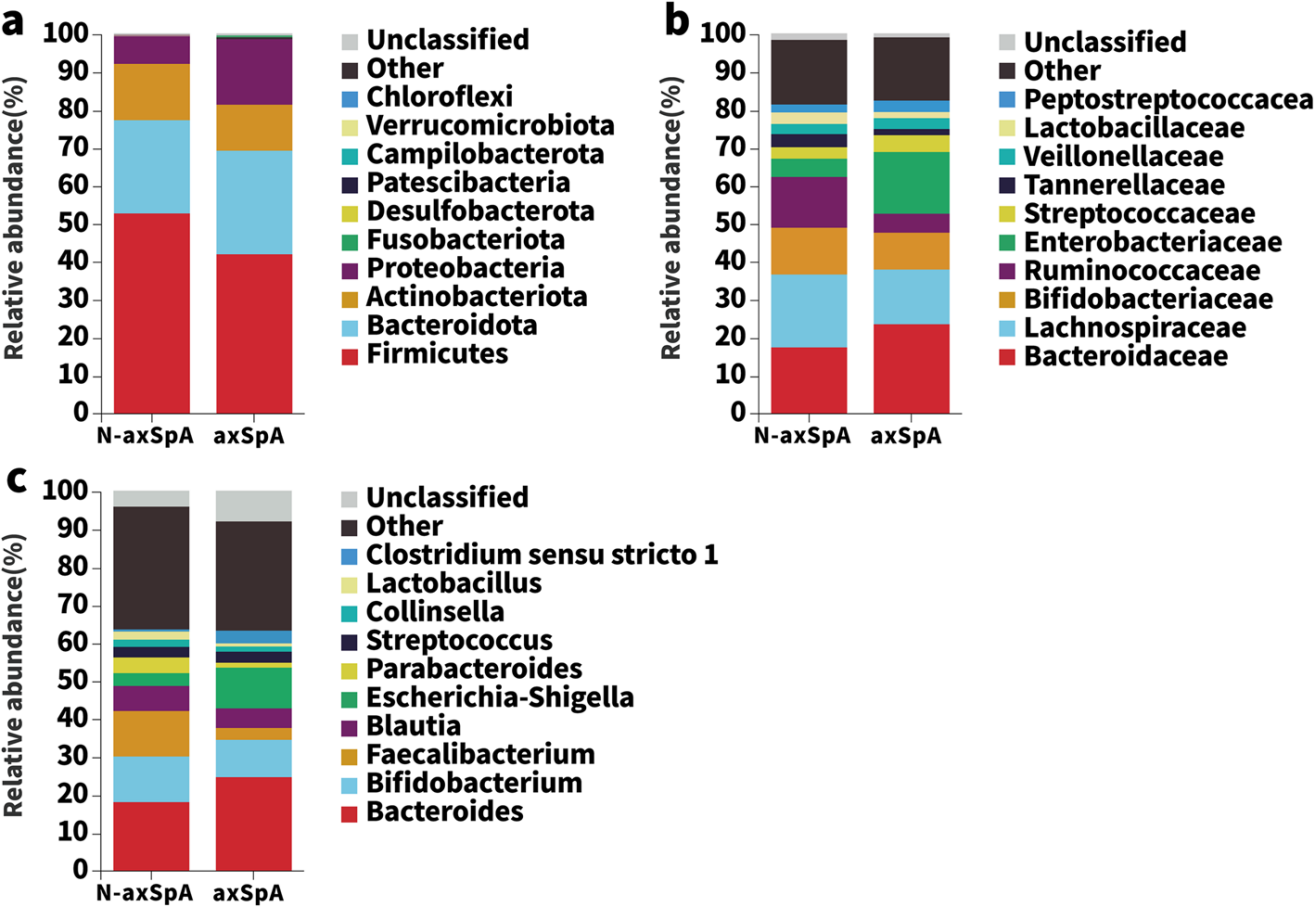
Stacked bar plots of the community compositions

The axSpA fecal samples yielded reads corresponding to 18 phyla, 130 families, and 206 genera. The non-axSpA samples yielded reads corresponding to 19 phyla, 104 families, and 175 genera. The gut microbiotas of the axSpA patients yielded significantly higher proportions of 18 taxa and lower proportions of 20 taxa compared with those of the non-axSpA patients. At the phylum level, axSpA patients had significantly higher proportions of Proteobacteria (7.55% vs 17.76%, *P*=0.023) and lower proportions of Firmicutes (52.65% vs 41.97%, *P*=0.019) than did non-axSpA patients (Fig. 4a). At the family level, axSpA patients had significantly higher proportions of Enterobacteriaceae (4.84% vs 16.12%, *P*=0.016) and lower proportions of Ruminococcaceae (14.01% vs 5.23%, *P*=0.004; Fig. 4b). At the genus level, axSpA patients had significantly higher proportions of *Escherichia_Shigella* (3.61% vs 10.32%, *P*=0.031) and lower proportions of *Faecalibacterium* (11.88% vs 2.84%, *P*=0.001; Fig. 4c).

**Fig. 5:**
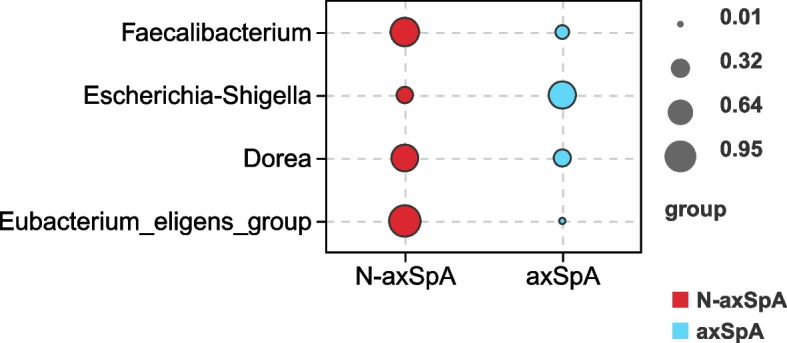
Bubble chart of the fecal flora indicator analysis

Indicator analysis revealed that *Escherichia_Shigella* (indicator value [IndVal]=0.74, *P*=0.007) was the more likely indicator species in the axSpA group, and *Faecalibacterium* (IndVal=0.81, *P*=0.002) was the more likely indicator species in the non-axSpA group (Fig. 5).

#### Functional prediction

**Fig. 6:**
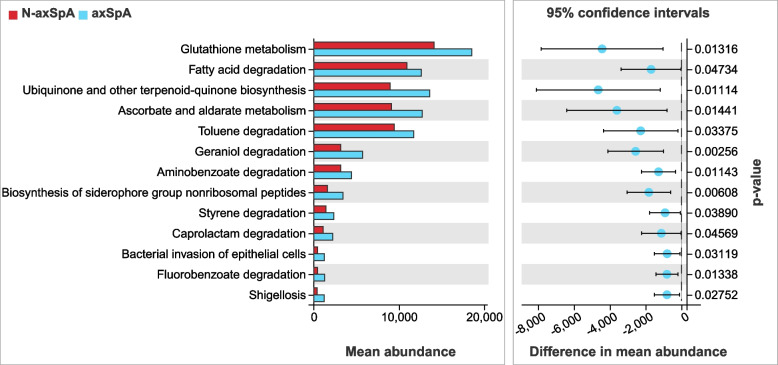
Metabolic pathway analysis of the fecal flora in patients with UC

KEGG pathway analysis of the OTUs was inferred using PICRUSt. Many biosynthetic and metabolic pathways were upregulated in the axSpA group compared with those of the non-axSpA group, including glutathione metabolism, fatty acid degradation, ubiquinone and other terpenoid-quinone biosynthesis, ascorbate and aldarate metabolism, geraniol degradation, and biosynthesis of siderophore group nonribosomal peptides (Fig. 6).

## Discussion

Of patients with IBD, 3%–25% will also develop axSpA [4–6]. In this study, 19.2% of patients with UC also had axSpA. The high prevalence of axSpA in patients with UC suggests that gastroenterologists should pay attention to early screening of extraintestinal joint manifestations in these patients and that patients with inflammatory low back pain should be differentiated from those with degenerative changes of the spine and promptly referred to rheumatologists for early diagnosis and treatment [12]. Genome-wide association analysis showed that patients with IBD carrying the HLA-B27 genotype had an increased risk of axSpA [13, 14]. Misfolding of the HLA-B27 molecule promotes endoplasmic reticular stress and triggers an unfolded protein response that stimulates interleukin (IL)-23/IL-17 production, which is closely related to the axSpA pathogenesis [15]. Prevalences of HLA-B27 positivity were 25%–78% in patients with IBD-associated AS and 7%–15% in patients with isolated sacroiliitis compared with 4%–8% in the general Asian population [13, 14]. In the current study, the HLA-B27 positivity rate in the concomitant axSpA group was 42.9%.

The intestinal flora plays an important role in maintaining bodily health, including participating in nutrient metabolism, maintaining intestinal permeability, and enhancing immune system function [16]. Dysbiosis has been correlated with development of many diseases, including IBD, colon cancer, diabetes, obesity, and AS [17–19]. Dysbiosis-induced loss of intestinal mucosal barrier integrity is thought to be an important causative factor for occurrence and recurrence of IBD and axSpA [20–22]. Intestinal barrier damage and increased intestinal permeability occur first in the preclinical pathogenesis of most IBDs and axSpA [9]. Flora antigens and intestinal immune cells can migrate through the damaged intestinal mucosal barrier to the joints (i.e., the gut-synovial axis), which in turn triggers axSpA [10, 14]. Decreased diversity and abundance in the intestinal flora have been reported in patients with axSpA and IBD; however, few studies have reported whether the structure of the fecal flora differs between IBD patients with and without axSpA. We found that beta diversities of the fecal flora differed between the axSpA and non-axSpA groups, which is consistent with the diversity analysis results of Sternes et al. [23]. Compared with those of the non-axSpA group, the Firmicutes, Ruminococcaceae*,* and *Faecalibacterium* abundances were decreased, and the Proteobacteria, Enterobacteriaceae, and *Escherichia_Shigella* abundances were increased in the axSpA group.

Similar to previous studies [24, 25], *Escherichia_Shigella* was more likely to be the indicator species of the axSpA group. *Escherichia* and *Shigella* are important pathogenic genera of Enterobacteriaceae and are mostly gram-negative bacilli whose cell membranes contain proinflammatory lipopolysaccharides associated with intestinal inflammation and intestinal mucosal barrier damage [26]. Adherent-invasive *Escherichia coli* (AIEC) is an important member of *Escherichia_Shigella* and encodes the large subunit of propylene glycol dehydratase. Viladomiu et al. [25] colonized a mouse model with AIEC enriched from the intestines of patients with Crohn’s disease accompanied by spondyloarthritis and found that AIEC used propylene glycol produced by fermentation of fucoidan as a carbon source to proliferate in the mucosal layer, thereby triggering inflammation. Furthermore, the propylene glycol dehydratase metabolite propionic acid and lipopolysaccharide synergistically stimulated IL-1β production by CX3CR1+ monocyte macrophages, induced systemic Th17 immune abnormalities, and promoted intestinal inflammation in mice. This inflammatory cascade depended on AIEC activating the catalytic activity of propylene glycol dehydratase [25, 27].

Current research on the gut flora is gradually transitioning from species to functional levels. PICRUSt2 analyses showed that the axSpA group was enriched in several biosynthetic and metabolic pathways compared with those of the non-axSpA group. These significantly different metabolic pathways may help clarify the specific pathological mechanisms by which gut flora affect UC-axSpA. Glutathione metabolism is an important pathway for resisting oxidative stress injury in various gram-negative bacteria, including *Escherichia coli* and *Pseudomonas aeruginosa* [28]. Geraniol inhibits IL-1β-induced expression of prostaglandin E2, cyclooxygenase 2, TNF-α and IL-6 by downregulating the PI3K/AKT/NF-κB and MAPK signaling pathways and exerts anti-inflammatory effects while inhibiting MMP-9 and ADAMTS-5 expression, reversing degradation of aggregated proteoglycans and type II collagen, and possessing antichondrogenic effects [29].

Iron carriers produced via the nonribosomal peptide synthase pathway are key virulence factors for many pathogenic bacteria, such as *Escherichia coli*, *Pseudomonas aeruginosa*, *Klebsiella pneumoniae*, and *Staphylococcus aureus* [30]. Drugs synthesized against iron carriers, such as vancomycin, mycopeptides, and cyclosporine, have been widely used. Thus, we hypothesized that the increased abundance of *Escherichia_Shigella* in the fecal flora of patients with concomitant axSpA can induce intestinal and arthritic inflammation by using iron carriers to acquire iron and compete with the host for iron uptake and for glutathione resistance to oxidative stress and activation of virulence gene expression [28, 30]. Conversely, geraniol, with its anti-inflammatory and antichondrodegradative properties, may ameliorate intestinal and joint damage in patients with concomitant axSpA.

This study had some limitations. The sample size was small, and some factors affecting axSpA may have been excluded; thus, the conclusions are somewhat limited. The disease characteristics of axSpA were not evaluated; we collected only the clinical characteristics of UC and serum inflammatory markers at baseline, and no intervention or post-treatment follow-up of the UC patients was conducted. Based on the findings from this preliminary study, we are currently conducting a multiple-center study to recruit more qualified IBD patients who have extraintestinal manifestations.

## Conclusion

In this study, 19.2% of patients with UC had axSpA. Beta diversity and community composition of the fecal flora differed significantly between the axSpA and non-axSpA groups. Changes in the relative abundances of *Escherichia_Shigella* may be related to axSpA incidence. Additionally, multiple biosynthetic and metabolic pathways were enriched in the axSpA group compared with those in the non-axSpA group. Identification of an inflammatory pathway between gut dysbiosis and musculoskeletal inflammation could revolutionize therapeutic approaches for axSpA.

### Supplementary Information

**Additional file 1: Supplementary Fig. S1.** Rarefaction curves.

## Data Availability

The datasets analysed during the current study are available in the NCBI repository, [BioProject ID: PRJNA1063212].
