# Supplementary material for: Clinical features and fecal microbiota characteristics of patients with both ulcerative colitis and axial spondyloarthritis
Source: BMC Gastroenterol. 2024 Jan 31;24:56. doi: 10.1186/s12876-024-03150-w (PMC10832282; doi:10.1186/s12876-024-03150-w)
Supplement: Supplementary file 1 — Additional file 1: Supplementary Fig. S1. Rarefaction curves. [file 12876_2024_3150_MOESM1_ESM.docx]

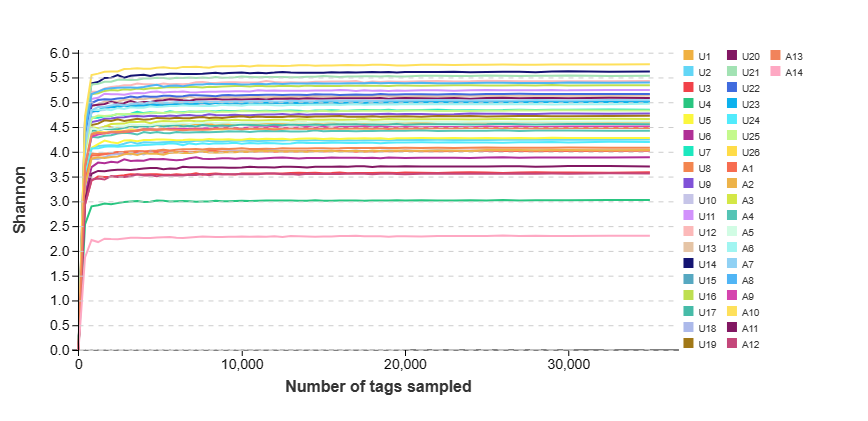


**Supplementary Fig. S1. Rarefaction curves**

The sample numbers A1 to A14 belong to the axSpA group, while the sample numbers U1 to U26 belong to the non-axSpA group. The graph has the number of randomly selected tags on the horizontal axis and the expected value of the Shannon index on the vertical axis. As the number of tags increases, the Shannon index gradually goes up. When the sequencing number reached 10,000 tags, the curve began to flatten out, indicating that the sequencing amount was sufficient to reflect most of the microbial information in the sample.
